# Psychoeducation for bipolar disorder and risk of recurrence and hospitalization – a within-individual analysis using registry data

**DOI:** 10.1017/S0033291719001053

**Published:** 2020-04

**Authors:** Erik Joas, Kristoffer Bäckman, Alina Karanti, Timea Sparding, Francesc Colom, Erik Pålsson, Mikael Landén

**Affiliations:** 1 Department of Psychiatry and Neurochemistry, Institute of Neuroscience and Physiology, The Sahlgrenska Academy at University of Gothenburg, Gothenburg, Sweden; 2 Mental Health Group, IMIM-Hospital del Mar-CIBERSAM, Barcelona-Catalonia, Spain; 3 Department of Medical Epidemiology and Biostatistics, Karolinska Institutet, Stockholm, Sweden

**Keywords:** Bipolar disorders, epidemiology, psychoeducation

## Abstract

**Background:**

The efficacy of psychoeducation for bipolar disorder has been demonstrated in clinical trials, but it is not known if the results translate into effectiveness in routine clinical practice. The aim was to determine the effectiveness of psychoeducation for bipolar disorder in a routine clinical setting.

**Method:**

We identified 2819 patients with at least three registrations in the Swedish Quality Assurance Register for Bipolar Disorder. Among those, 402 had not been exposed to psychoeducation at the first visit, but received psychoeducation during any of the following registrations. Using within-individual analyses, the risk of recurrence after having received psychoeducation was compared with the risk prior to psychoeducation.

**Results:**

In adjusted within-individuals comparisons, periods after psychoeducation was associated with decreased risks of any recurrence [odds ratio (OR) 0.57, 95% CI 0.42–0.78], (hypo-)manic or mixed episodes (OR 0.54, 95% CI 0.39–0.76), depressive episodes (OR 0.63, 95% CI 0.47–0.86), and inpatient care (OR 0.54, 95% CI 0.33–0.86) relative to periods prior to psychoeducation. There was no association with rates of involuntary sectioning or suicide attempts.

**Conclusions:**

The results suggest that psychoeducation for bipolar disorder reduces the risk of mood episodes and inpatient care also when implemented in routine clinical practice.

## Introduction

Pharmacological maintenance treatment is the cornerstone in bipolar disorder management. Unfortunately, relapse rates remain high despite mood stabilizing treatment (Pallaskorpi *et al*., 2015). Psychoeducation programs are adjunctive interventions that complement pharmacological treatment with the aim to further reduce illness burden and recurrence. Programs are believed to achieve this not only by increasing patients' knowledge of their disorder, but also by changing key attitudes and behaviors toward improved medication adherence and a healthier lifestyle. Programs include education about the risk of recurrence having a chronic condition, treatment options, the risks of drugs and alcohol, as well as the importance of sleep, routines, and healthy habits. Programs also include training to identify personal early warning signs of an imminent episode, and training to manage symptoms. There are several psychoeducational programs for bipolar disorder but only a few have been evaluated in randomized controlled trials. Even though the length of psychoeducational programs vary – some programs are completed within 6 weeks (Parikh *et al*., 2012) other last up to 6 months (Colom *et al*., 2003*a*) – they include similar key ingredients.

Psychoeducation has been shown to reduce relapse rate (Bond and Anderson, 2015), increase adherence to medication (Colom *et al*., 2005; Eker and Harkin, 2012), and improve social functioning (Perry *et al*., 1999). Even though psychoeducation is now recommended in several bipolar management guidelines (Yatham *et al*., 2009; Goodwin *et al*., 2016), concern has been raised that the purported effect of psychoeducation on relapse prevention (Bond and Anderson, 2015) is being too reliant on two pivotal studies (Colom *et al*., 2003*a*, *b*). In fact, a recent study failed to demonstrate an effect on relapse except in a sub-group of patients with few previous episodes (Morriss *et al*., 2016).

The gold standard to prove efficacy of an intervention is randomized controlled trials (RCTs). But results from RCTs are not readily generalizable to routine clinical practice. This is because RCTs often employ strict inclusion and exclusion criteria generating study populations that may differ from patients seen in routine clinical practice. Psychoeducation trials have enrolled patients from academic centers (Parikh *et al*., 2012) and excluded people with comorbidities (Colom *et al*., 2003*a*; de Barros Pellegrinelli *et al*., 2013). It is therefore important to complement RCTs with observational studies to evaluate the effectiveness of interventions in routine clinical practice. Although some observational and non-randomized studies corroborate the positive results of psychoeducation (Michalak *et al*., 2005; Tidemalm *et al*., 2007; Candini *et al*., 2013), observational studies are hampered by confounding-by-indication. This is because the indication for the intervention might be correlated with the outcome. For example, attending a psychoeducational program has been shown to be associated with better adherence *prior* to the intervention (Cakir *et al*., 2009), which might bias the results in favor of psychoeducation.

The aim of this study was to estimate the effectiveness of psychoeducation for bipolar disorder in a routine clinical setting. To partly circumvent the problem of confounding by indication we used a within-individual design that controls for confounding caused by differences in time-stationary covariates.

## Method

### Sample

Data were obtained from the Swedish quality assurance register for bipolar disorder (BipoläR) (Karanti *et al*., 2015; Karanti *et al*., 2016). BipoläR was established in Sweden in 2004 with the aim to improve the quality of the care of bipolar patients in Sweden. The register contains individualized data on patients’ bipolar disorder type (I, II, NOS, cyclothymia, or schizoaffective disorder bipolar type), demographics, interventions, and outcomes. Data are collected by staff at the psychiatric clinics (e.g. psychiatrists, psychiatric nurses, or psychologists) and entered into a web-based application. Registering units include both private and public psychiatric outpatient health care units, and BipoläR covers most health care regions in Sweden. The study was approved by the Regional Ethics Committee in Gothenburg.

Patients can be included in the register at any time, at which a baseline registration is completed. Patients are then expected to be followed-up annually. By the time of data extraction for this study in late 2013, the database included baseline data on 12 850 individuals with 31 470 unique visits (baseline registrations + annual follow-ups). The number of individual visits (baseline registration + annual follow-ups) varied between 1 and 10.

Registrations at baseline and annual follow-ups include data on, for example, the number of affective episodes (depressive, manic, hypomanic, or mixed episodes), psychiatric hospital admissions, suicide attempts or self-harm, medication, and a split global assessment of functioning (GAF) scale using a 100 point scale to assess the patients function and symptom burden, where we used the symptom dimension for this study (Pedersen *et al*., 2007). The BipoläR register included approximately 20% of the total number of bipolar disorder patients in Sweden (data from the National Patient register) during the study period. The sex distribution in BipoläR is on par with the whole bipolar disorder population. Patients who receive inpatient care but fail to establish follow-up contact with outpatient clinics will, however, not be captured in BipoläR.

### Measurements

At baseline (the first time a patient is entered in the register), the question regarding psychoeducation read: *Has the patient obtained psychoeducation?* At the annual follow-up registrations, the question read: *Has the patient received psychoeducation during the last 12 months?* As treatment periods, we used all periods after the registration at which it was first documented that the patient had received psychoeducation. A formal definition of psychoeducation was not introduced in BipoläR until 2013: ‘*Psychoeducation should be a pre-defined program that comprise education about the disorder, include strategies to cope with the disorder, and give the possibility for exchange of experiences*.’ Even though no formal definition was available prior to 2013, a survey of psychoeducation programs reported to BipoläR concluded that Swedish psychoeducation programs on average comprised of six 2-h sessions, and that group setting was most common (Askland and Ahmad Sadik, 2016).

Outcome variables were admissions to psychiatric in-patient care, involuntary sectioning, suicide attempts or self-harm, depressive episodes, and (hypo-) manic or mixed episodes. Data on mood episodes and other outcomes during the 12 months preceding the annual follow-up interviews were obtained retrospectively by treating clinicians with access to medical charts.

### Statistical analysis

To eliminate unmeasured time-stationary confounding, we analyzed data using a within-individual method in which the individual serves as his/her own control. This is important because patients are not randomly assigned to receive psychoeducation in clinical routine. For example, patients with more severe bipolar disorder could be more likely to receive psychoeducation, or vice versa. Either way, such bias would introduce confounding by indication, i.e. confounding caused by the fact that patients have been selected for a specific treatment based on a higher or lower propensity for a certain outcome. The within-individual design used in this study was recently employed to evaluate the effectiveness of mood-stabilizing drugs in a naturalistic setting (Joas *et al*., 2017), and the risk of treatment-emergent switch to mania following antidepressant or central stimulant treatments (Viktorin *et al*., 2014; Viktorin *et al*., 2017).

**Fig. 1.**
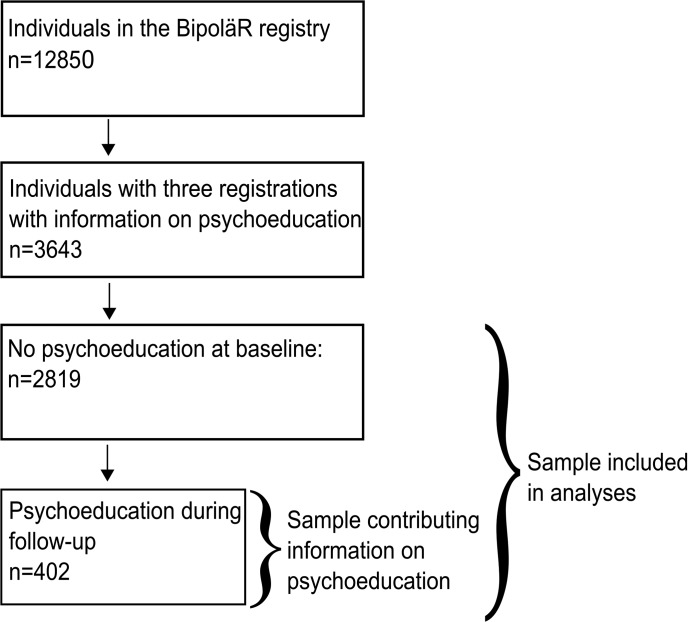
Sample selection.

**Fig. 2.**
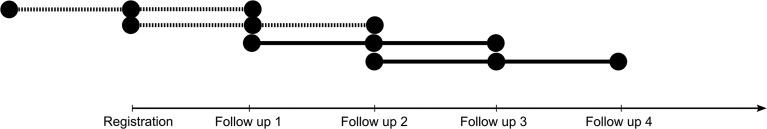
Schematic view of the study design visualized using one individual's participation. Each line represents a time interval. Note that the number of follow-ups may vary among individuals but includes a minimum of two. The dotted lines indicate non-treatment intervals. Full lines indicate a treatment period, i.e. when the individual had received patient education. The registration or follow-up occurs at the mid-section of each line. Data on completed psychoeducation is collected for the 12 month period preceding the follow-up, whereas the outcome measures are measured at the following visit regarding outcomes 12 months before that visit. Notice the timing of outcome information from one segment might overlap with information on psychoeducation for the following segment. This has been addressed in a sensitivity analysis (Figure S1 in the online data supplement).

We divided the data into time periods, each with a baseline measurement indicating whether the person had or had not received psychoeducation, followed by the subsequent measurement indicating the outcome (for instance if the person had suffered from depressive periods during the last 12 months). The sample was selected as follows: first, we excluded follow-ups that occurred earlier than 0.75 years or later than 2.0 years after the preceding registration in order to limit variability in time between visits. Second, we excluded those who had received psychoeducation at baseline, i.e. prior to the first assessment. This means that patients were treatment-naïve with respect to psychoeducation when entering the study. Third, the first three measurements for each person had to include information on psychoeducation. This is because we needed three visits to construct at least two time intervals. The final sample consisted of 2819 individuals. Within this group, 402 persons had psychoeducation at any follow-up and could contribute with information on the effectiveness of psychoeducation. Figure 1 displays a schematic view of the sample selection. Figure 2 shows a schematic view of the analysis plan. Data were analyzed using conditional logistic regression (Allison, 2006), stratified on individuals. Although only individuals with a changing status of psychoeducation (*N* = 402) could provide information directly on the effect of psychoeducation, other individuals (*N* = 2417) provide information on the effects of confounding variables such as age and GAF. Furthermore, the within-individual models only make use of individual variation, meaning that only individuals that change status on outcome variables provide information. These numbers are given for each outcome in tables throughout.

The following outcome variables were analyzed in relation to psychoeducation: any mood episode, (hypo-) manic or mixed episode, depressive episode, inpatient care, involuntary sectioning, and self-harm or suicide attempts. We analyzed the effect of psychoeducation in unadjusted models and models adjusted for the symptom dimension of the Global Assessment of Functioning (GAF) scale, age, and treatment with mood stabilizers (lithium or anticonvulsants). Listwise deletion was employed for intervals with missing values. The results are presented as odds ratios (ORs) with 95% CI, and we used *p* < 0.05 as the threshold for statistical significance. Pearson's χ^2^ and *t* tests were used to test differences in Table 1.

**Table 1.** Characteristics of the study sample

	No psychoeducation during the study period (*N* = 2417)	Psychoeducation during the study period (*N* = 402)	*p*	Missing
GAF-symptom at baseline, mean (s.d.)	66.9 (13.0)	64.4 (12.4)	0.001	130
Number of intervals during follow-up, mean (s.d.)	3.20 (1.23)	3.57 (1.31)	<0.001	
Age at baseline, mean (s.d.)	52.2 (15.1)	43.1 (13.3)	<0.001	
Male, *N* (%)	1010 (41.8)	129 (32.1)	<0.001	
Bipolar diagnosis at baseline, *N* (%)			0.068	39
Type I	1066 (44.8)	182 (45.7)		
Type II	798 (33.5)	148 (37.2)		
UNS	424 (17.8)	61 (15.3)		
Schizoaffective disorder	94 (3.9)	7 (1.8)		
Mood stabilizing treatment at baseline, *N* (%)	2146 (88.8)	356 (88.6)	0.960	
Episode during follow up, *N* (%)	1575 (66.6)	348 (87.9)	<0.001	59
Age at first symptom, *N* (%)			<0.001	1085
0–7 years	47 (3.2)	17 (6.3)		
8–11 years	68 (4.6)	21 (7.7)		
12–17 years	385 (26.3)	93 (34.3)		
18–24 years	370 (25.3)	66 (24.4)		
25-years	593 (40.5)	74 (27.3)		

Although our main mode of analysis was to explore within-individual difference, we also present between-individual models to show differences between the group that received psychoeducation and those that did not. As there were several time intervals per person, we used a logistic GEE model (Halekoh *et al*., 2006) with an exchangeable correlation structure to account for the correlation between observations on the same individual. These models were adjusted for mood stabilizing medication, GAF-symptom, age, and sex.

### Sensitivity- and sub-analyses

Four sensitivity analyses and sub-analyses were conducted to test the robustness of our results. First, it could be possible that the effect of psychoeducation attenuates over time. We therefore conducted a sensitivity analysis by using only the first interval with psychoeducation while excluding subsequent periods. Second, patients' status in the interval just before psychoeducation might be associated with why the person received psychoeducation. We thus conducted a sensitivity analysis where we excluded the segment immediately before psychoeducation. As information on completed psychoeducation was obtained from the same registration as outcomes during the previous segment, there is a possible overlap in the last segment considered to be without psychoeducation. We therefore removed the segment before psychoeducation in a sensitivity analysis to eliminate this possibly ambiguous time interval. Third, the psychoeducation might have occurred early in a time period and influence outcomes at the same registration where psychoeducation was first registered. We therefore computed time intervals where we used measures of psychoeducation and outcomes from the same visits. However, this means that the outcome could have occurred before the event. Therefore, we also included a fourth sensitivity analysis where we excluded such ambiguous observations. Sensitivity analyses #3 and #4 were only computed with the outcome variable ‘relapse in any mood episode’ as the question regarding relapses during the last 12 months at the baseline registration encompassed all relapses. For a schematic view of the sensitivity- and sub-analyses, see Figure S1 in the online Supplementary material.

## Results

The characteristics of the study sample are presented in Table 1. Only diagnostic subgroup and mood stabilizing medication at baseline were significantly different between the two groups. For descriptive purposes, we calculated the percentage of individuals with any mood episode during the study periods for the two groups, adjusted for GAF-symptom, mood stabilizing treatment, sex, and age at baseline. Any mood episode occurred in 86.5% of the psychoeducation group and 69.4% in the no psychoeducation group. Notably, these numbers include relapses during the whole study period, including periods before as well as after psychoeducation.

Table 2 shows the results from the main conditional logistic regression analyses. It should be noted that the fully adjusted models have fewer observations than the unadjusted models due to missing data on covariates.

**Table 2.** Effect of psychoeducation on different outcomes using conditional logistic regression with separate strata for each individual (total number of individuals = 2819; individuals receiving psychoeducation (PE) during follow-up = 402; time intervals = 9161)

Outcome	OR	(95% CI)	*p* value	Missing^a^	*N* with change on outcome	*N* with change on outcome + PE	aOR^b^	(95% CI)	*p* value	Missing^a^	*N* with change on outcome	*N* with change on outcome + PE
All relapses	0.48	(0.36–0.64)	<0.01	65	1211	213	0.57	(0.42–0.78)	<0.01	591	1152	207
(Hypo-) manic or mixed episodes	0.49	(0.36–0.66)	<0.01	109	983	192	0.54	(0.39–0.76)	<0.01	634	917	183
Depressive episodes	0.53	(0.4–0.7)	<0.01	109	1167	217	0.63	(0.47–0.86)	<0.01	634	1099	208
Suicide attempts or self-harm	0.62	(0.3–1.29)	0.20	109	158	33	1.22	(0.54–2.76)	0.64	635	146	32
Inpatient care	0.51	(0.33–0.78)	<0.01	63	511	92	0.54	(0.33–0.86)	0.01	591	484	89
Involuntary sectioning	0.68	(0.38–1.24)	0.21	115	223	49	0.66	(0.34–1.3)	0.23	640	213	45

a The number of time intervals with missing data.

b Adjusted for age, mood stabilizing treatment, and GAF-symptom.

Psychoeducation was significantly associated with a decreased rate of any mood episode, (hypo-) manic episodes, mixed episodes, depressive episodes, and inpatient care in the unadjusted as well as in the adjusted analyses. Importantly, only individuals with a changing status on psychoeducation (*N* = 402) contributed information directly on the effect of psychoeducation, other individuals (*N* = 2417) provide information on the effects of confounders such as age and GAF.

The first sensitivity analysis (Table S1 in online data supplement), which only used the first time segment after receiving psychoeducation, showed similar results. The results were also similar in the unadjusted second sensitivity analysis (online Supplementary Table S2), which applied a washout period prior to the first instance of psychoeducation, although the estimates were attenuated in the adjusted analysis and not statistically significant except for inpatient care. Sensitivity analyses #3 and #4 (online Supplementary Table S3) showed similar results as the main analysis for all relapses.

For descriptive purposes, we also calculated between-group differences (online Supplemental Table S4) using a GEE model (Halekoh *et al*., 2006). In these between-group estimates, neither relapse rate nor inpatient care differ between the psychoeducation and no-psychoeducation groups.

## Discussion

We used a within-individual design to study the effectiveness of psychoeducation in bipolar disorders in routine clinical practice. Our sample consisted of 2819 subjects of whom 402 individuals received psychoeducation during the follow-up. The results suggest that psychoeducation reduces the risk of mood episodes and hospital admission in routine clinical practice, but we found no evidence for reduced rates of involuntary sectioning, or suicide attempts and self-harm. The sensitivity analyses were chiefly consistent with the main analyses. This observational study is an important complement to previous RCTs suggesting that psychoeducation is effective also when implemented on a large scale in routine clinical practice.

Our findings are in line with a recent meta-analysis on RCTs of psychoeducation that showed a protective effect on any relapse as well as on manic relapse (Bond and Anderson, 2015). The effect on depressive relapse was only evident when considering programs that delivered the treatment in a group setting. By contrast, however, the latest large scale RCT of psychoeducation did not show an effect of psychoeducation except for those with a low number of previous episodes (Morriss *et al*., 2016). Notably, this randomized effectiveness study used an active control, which for obvious reason cannot be used outside a trial setting. Hence, our study gives no information on the active components of psychoeducation.

In most prior RCT studies of psychoeducation, individuals with bipolar II disorder were underrepresented, which means that results apply principally to bipolar I disorder. The current study includes almost equal numbers of subjects with bipolar disorder type I and II (45.6% *v.* 37.3%), and thus adds to the literature on the effect of psychoeducation in bipolar II disorder. Our results suggest that psychoeducation is effective in both bipolar type I and II disorder, which is in line with Colom *et al*.'s *post-hoc* analysis in a subgroup of patients with bipolar type II (Colom *et al*., 2009).

Previous RCTs had well-defined programs of psychoeducation. By contrast, the quality register BipoläR did not provide a definition of psychoeducation during the study period, which means that this study does not include information on the length or setting of the definition of the psychoeducational program. However, a recent survey of psychoeducational programs for bipolar disorder in Sweden found that psychoeducational programs on average consisted of six 2-h sessions, and that group setting was most common (Askland and Ahmad Sadik, 2016). Even though this is briefer than for example the Barcelona program (Colom *et al*., 2003*a*), a study found that a short program showed comparable effectiveness to 20 sessions of CBT (Parikh *et al*., 2012). Another study demonstrated improved adherence in a 6-week psychoeducation program (Eker and Harkin, 2012). Taken together, these results suggest that briefer psychoeducation curriculums are also effective and might provide an even more cost-effective alternative to more comprehensive programs, which have previously been shown to be cost-effective (Scott *et al*., 2009). This is important because more extensive programs, e.g. the Barcelona model, not only requires staff training but are also time consuming, which raises barriers to psychoeducation access. Further, smaller general psychiatric outpatient units might not have a sufficient patient base to make the effort worthwhile. Developing a web-based education is one option that would remove some barriers to access.

In previous clinical trials, psychoeducation has been delivered by educators with varying clinical experience and professional backgrounds: experienced psychologist (Colom *et al*., 2003*a*), psychologist with little clinical experience (Perry *et al*., 1999), or experienced psychiatric staff including both psychologists, nurses, and psychiatrist (Parikh *et al*., 2012). In our study, psychoeducation was probably most commonly delivered by nurses, followed by psychiatrists and psychologists (Askland and Ahmad Sadik, 2016).

We did not find an effect of psychoeducation on involuntary sectioning and suicide and self-harm. This could be due to the low number of events and limited power, but it could also be that these severe outcomes are not prevented by psychoeducation. Indeed, although suicidality and self-harm and involuntary treatment are addressed in some longer psychoeducational programs, these topics are not always included in shorter programs given by local clinics in Sweden. We also analyzed our data with between-individual models that did not show any association between psychoeducation and the selected outcomes. However, between-individual models are susceptible to confounding-by-indication, whereas the within-individual model control for time-stationary confounders.

### Strengths and limitations

The major strength of this study is the large sample of persons with bipolar disorder treated in a natural setting. The results are therefore generalizable to bipolar disorder patients in routine clinical practice. The study should, however, be interpreted with several limitations in mind. (i) The study is observational and not randomized, which precludes causal conclusions on the effect of psychoeducation. Females and younger patients were overrepresented in psychoeducation programs. The survey of psychoeducational programs in Sweden reported that almost half of responding clinics actively prioritized patients for psychoeducation, the basis of which varied between clinics, but included newly diagnosed patients, young patients, and patients with frequent relapses (Askland and Ahmad Sadik, 2016) reflecting clinical decisions backed by recent evidence (Morriss *et al*., 2016). Conversely, clinics reported that they also had criteria for patients less fit for psychoeducation, such as ongoing substance abuse, not being euthymic, lack of insight, personality disorder, numerous previous episodes, and low cognitive ability (Askland and Ahmad Sadik, 2016). To circumvent this potential confounding by indication, we used a within-individual design. This method controls for confounding caused by differences in time-stationary covariates, such as sex, genetic makeup, premorbid history, and lifetime severity of the disorder. We also adjusted for GAF, age, and mood-stabilizing drug use as time-varying confounders. Nevertheless, unmeasured time-varying confounders may still have influenced the outcome. (ii) Given that we rely on annual reports, the timing of intervention and measures of outcomes might in some cases overlap. We therefore conducted a sensitivity analysis where we removed the segment immediately before psychoeducation. In this analysis, decreased risk of inpatient care after psychoeducation was the only significant finding. This analysis comprised fewer observations due to the exclusion of many time-intervals. Hence, lower statistical power probably plays a large part. But the point estimates were also somewhat attenuated when compared to the primary analysis and it cannot be excluded that decreased time-varying confounding might play a role: If severity of illness would be associated with receiving psychoeducation, removing the segment immediately prior to psychoeducation would decrease differences in outcome before and after psychoeducation. There is also the possibility that psychoeducation took place before the outcome due to overlap, but such bias would rather lead to null results in the primary analysis. (iii) Finally, the lack of a formal definition of psychoeducation in the BipoläR questionnaire is a limitation as it reduces the specificity of the exposure variable.

## Conclusion

Using a within-individual design that controls for a time-stationary confounding, we show that psychoeducation for bipolar patients in a routine clinical setting decreases the risk of mood new episodes and inpatient care. Although not all sensitivity analyses were entirely concurrent with the main results, the results taken together suggest that findings from previous efficacy clinical trials translate to effectiveness in routine clinical practice.

## Supplementary material

For supplementary material accompanying this paper visit https://doi.org/10.1017/S0033291719001053. click here to view supplementary material
