# Supplementary material for: Psychoeducation for bipolar disorder and risk of recurrence and hospitalization – a within-individual analysis using registry data
Source: Psychol Med. 2019 May 6;50(6):1043–9. doi: 10.1017/S0033291719001053 (PMC7191782; doi:10.1017/S0033291719001053)
Supplement: Supplementary file 1 [file S0033291719001053sup001.docx]

Online data supplement

**Title:** Psychoeducation for bipolar disorder and risk of recurrence and hospitalization – a within-individual analysis using registry data

**Authors:** Erik Joas^1^, M.Sc, Kristoffer Bäckman^1^, M.Sc, Alina Karanti^1^, M.D, Timea Sparding^1^, M.Sc , Francesc Colom^2^ PsyD, Ph.D, Erik Pålsson^1^, Ph.D , Mikael Landén^1, 3^, M.D, Ph.D

^1^Institute of Neuroscience and Physiology, University of Gothenburg, Sweden,

^2^Mental Health Group, IMIM-Hospital del Mar-CIBERSAM, Barcelona-Catalonia, Spain

^3^ Department of Medical Epidemiology and Biostatistics, Karolinska Institutet, Stockholm, Sweden

| Table S1. Effect of psychoeducation on different outcomes. Sensitivity analysis #1^1^ (totalt number of individuals = 2,819, individuals receiving psychoeducation during follow-up = 402, time intervals = 8,716) | | | | | | | | | | | | |
| --- | --- | --- | --- | --- | --- | --- | --- | --- | --- | --- | --- | --- |
| Outcome | OR | (95% CI) | p-value | Missing^2^ | N with change on outcome | N with change on outcome + PE | aOR^1^ | (95% CI) | p-value | Missing^2^ | Nr with change on outcome | Nr with change on outcome + PE |
| All relapses | 0.62 | (0.45, 0.86) | <0.01 | 61 | 1156 | 158 | 0.68 | (0.48, 0.96) | 0.03 | 576 | 1097 | 152 |
| (Hypo-) manic or mixed episodes | 0.62 | (0.44, 0.87) | 0.01 | 103 | 935 | 144 | 0.64 | (0.44, 0.92) | 0.02 | 617 | 870 | 136 |
| Depressive episodes | 0.61 | (0.44, 0.84) | <0.01 | 103 | 1115 | 165 | 0.68 | (0.48, 0.96) | 0.03 | 617 | 1049 | 158 |
| Suicide attempts or self-harm | 0.82 | (0.37, 1.82) | 0.63 | 100 | 152 | 27 | 1.29 | (0.54, 3.06) | 0.56 | 615 | 140 | 26 |
| Inpatient care | 0.48 | (0.29, 0.82) | 0.01 | 58 | 484 | 65 | 0.47 | (0.27, 0.82) | 0.01 | 574 | 458 | 63 |
| Involuntary sectioning | 0.72 | (0.36, 1.46) | 0.37 | 104 | 209 | 35 | 0.57 | (0.26, 1.25) | 0.16 | 618 | 200 | 32 |
| 1) In contrast to the main model, the time intervals after the first time interval with psychoeducation were removed.  2) Adjusted for age, mood stabilizing treatment, and GAF-symptom.  3) Time intervals with missing data | | | | | | | | | | | | |
| Table S2. Effect of psychoeducation on different outcomes. Sensitivity analysis #2^1^ (total number of individuals = 2,668, individuals receiving psychoeducaton during follow-up that also have a time-interval without pychoeducation = 132, time intervals = 8,539) | | | | | | | | | | | | |
| Outcome | OR | (95% CI) | p-value | Missing^2^ | N with change on outcome | N with change on outcome + PE | aOR^1^ | (95% CI) | p-value | Missing^2^ | Nr with change on outcome | Nr with change on outcome + PE |
| All relapses | 0.51 | (0.31, 0.86) | 0.01 | 64 | 1117 | 68 | 0.67 | (0.39, 1.16) | 0.16 | 576 | 1058 | 66 |
| (Hypo-) manic or mixed episodes | 0.60 | (0.36, 0.99) | 0.05 | 98 | 903 | 68 | 0.66 | (0.37, 1.16) | 0.15 | 610 | 838 | 62 |
| Depressive episodes | 0.53 | (0.32, 0.87) | 0.01 | 98 | 1069 | 71 | 0.76 | (0.44, 1.3) | 0.31 | 610 | 1002 | 66 |
| Suicide attempts or self-harm | 0.39 | (0.07, 2) | 0.26 | 104 | 139 | 8 | 0.59 | (0.06, 5.52) | 0.64 | 616 | 126 | 7 |
| Inpatient care | 0.33 | (0.14, 0.78) | 0.01 | 58 | 476 | 28 | 0.34 | (0.13, 0.86) | 0.02 | 572 | 450 | 27 |
| Involuntary sectioning | 0.87 | (0.29, 2.6) | 0.81 | 110 | 200 | 14 | 0.63 | (0.16, 2.41) | 0.50 | 621 | 191 | 11 |
| 1) In contrast with the main model, the time interval right before the first report of psychoeducation was removed to avoid overlap.  2) Adjusted for age, mood stabilizing treatment, and GAF-symptom.  3) Time intervals with missing data | | | | | | | | | | | | |

| Table S3. Effect of psychoeducation on different outcomes. Sensitivity analysis #3^1^ and #4^2^. | | | | | | | | | | | | |
| --- | --- | --- | --- | --- | --- | --- | --- | --- | --- | --- | --- | --- |
| Outcome | OR | (95% CI) | p-value | Missing^2^ | N with change on outcome | N with change on outcome + PE | aOR^1^ | (95% CI) | p-value | Missing^2^ | Nr with change on outcome | Nr with change on outcome + PE |
| All relapses, sensitivity analysis 3 | 0.36 | (0.28, 0.45) | <.001 | 159 | 2082 | 340 | 0.56 | (0.43, 0.74) | <.001 | 953 | 1988 | 328 |
| All relapses, sensitivity analysis 4 | 0.29 | (0.22, 0.39) | <.001 | 154 | 2006 | 232 | 0.5 | (0.36, 0.71) | <.001 | 925 | 1914 | 223 |
| 1) Analyses with psychoeducation and outcomes measured concurrently (total number of individuals = 4,515, individuals receiving psychoeducation during follow-up = 668, time intervals = 15,465)  2) Analyses with psychoeducation and outcomes measured concurrently with the interval just before receiving psychoeducation removed to avoid overlap (total number of individuals = 4,334, individuals receiving psychoeducation during follow-up = 408, time intervals = 14,616)  3) Adjusted for age, mood stabilizing treatment, and GAF-symptom. | | | | | | | | | | | | |

| Table S4. A between-group analysis on the effect of psychoeducation on different outcomes using GEE logistic regression with exchangeable correlation structure (total number of individuals=2,819; number of individuals receiving psychoeducation during follow-up=402; number of time intervals=9,161). | | | | | | | | |
| --- | --- | --- | --- | --- | --- | --- | --- | --- |
| Outcome | OR | (95% CI) | p-value | Missing^2^ | aOR^1^ | (95% CI) | p-value | Missing^2^ |
| All relapses | 0.94 | (0.8, 1.1) | 0.44 | 65 | 0.93 | (0.79, 1.1) | 0.39 | 591 |
| (Hypo-) manic or mixed episodes | 0.95 | (0.79, 1.15) | 0.60 | 109 | 0.92 | (0.76, 1.11) | 0.38 | 634 |
| Depressive episodes | 0.93 | (0.79, 1.09) | 0.38 | 109 | 0.91 | (0.77, 1.08) | 0.3 | 634 |
| Suicide attempts or self-harm | 1.07 | (0.68, 1.69) | 0.77 | 109 | 0.93 | (0.6, 1.46) | 0.77 | 635 |
| Inpatient care | 0.95 | (0.73, 1.24) | 0.71 | 63 | 0.94 | (0.73, 1.23) | 0.67 | 591 |
| Involuntary sectioning | 1.03 | (0.65, 1.62) | 0.91 | 115 | 0.88 | (0.55, 1.39) | 0.57 | 640 |
| 1) Adjusted for age, mood stabilizing treatment, sex, and GAF-symptom.  2) The number of time intervals with missing data | | | | | | | | |


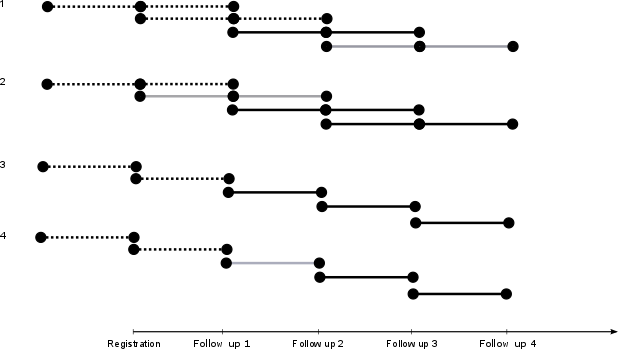


Figure S1.

Schematic view of sensitivity analyses visualized using one individual’s theoretical participation in the register. Note that the number follow-ups may vary among individuals. Each line symbolizes a time interval; the dashed lines indicate non-treatment intervals; full lines indicate a treatment period. The registration and follow-up registrations occurs at the mid-section of each line. Information on psychoeducation is collected for the 12-month period prior to the visit, whereas the information on the outcome measures (in sensitivity analysis 1 and 2) are measured at following visit concerning clinical outcomes 12 months prior to that visit. Notice the overlap between information on outcome from one segment and the information on psychoeducation for the following segment.

1. In this analysis, we excluded those intervals (marked in grey) that came after the first interval with psychoeducation, in order to study if the effect of psychoeducation is strengthened when only measuring the outcomes closest to the first instance of psychoeducation.

2. To avoid potential overlap between the first interval with psychoeducation and the outcomes of the previous interval, the interval before the first interval with psychoeducation was removed (marked in grey).

3. In this sensitivity analysis, we used the responses of outcomes and psychoeducation (+ confounders) measured at the same time.

4. In this sensitivity analysis, we used the same design as in sensitivity analysis 3. However, as psychoeducation and outcomes were measured at the same time, there is an uncertainty whether the psychoeducation was given before or after the outcomes at the first interval measuring psychoeducation. Therefore, this interval was removed in this analysis (marked in grey).
